# Efficiency and management factors: finding the balance in Thalassaemia care centres

**DOI:** 10.1186/s13561-021-00351-x

**Published:** 2022-01-26

**Authors:** Asrul Akmal Shafie, Noor Syahireen Mohammed, Kok Fong See, Hishamshah Mohd Ibrahim, Jacqueline Hui Yi Wong, Irwinder Kaur Chhabra

**Affiliations:** 1 Discipline of Social & Administrative Pharmacy, School of Pharmaceutical Sciences, Universiti Sains Malaysia, Pulau Pinang, Malaysia; 2 Clinical Research Centre, Hospital Sultanah Bahiyah, Kedah, Malaysia; 3 Economics Programme, School of Distance Education, Universiti Sains Malaysia, Pulau Pinang, Malaysia; 4 Department of Operations and Information Systems, Manning School of Business, University of Massachusetts at Lowell, MA, USA; 5 Deputy Director General’s (Research & Technical Support), Ministry of Health Malaysia, Putrajaya, Malaysia; 6 Pharmacy Department, Hospital Kuala Lumpur, Kuala Lumpur, Malaysia

**Keywords:** Thalassaemia treatment, Technical efficiency, Management factors, Data envelopment analysis, Malaysia

## Abstract

**Background:**

Optimizing efficiency has become increasingly critical with the growing demand for finite healthcare resources driven by population growth and an ageing society. Hence, policymakers are urgently finding more efficient ways to deliver health services. Thalassemia is a complex inherited blood disorder with significant prevalence in Malaysia. The high number of patients put substantial strain on the healthcare system. This study aims to evaluate the technical efficiency of thalassaemia care centres throughout Malaysia and the determinants that affect the efficiency.

**Method:**

Data from 30 public hospitals with thalassaemia care centres were collected. A double bootstrap data envelopment analysis (DEA) approach is used with the assumption of input-oriented and variable-to-scale DEA models to generate technical efficiency scores. Bootstrap truncated regression was later conducted to identify the factors affecting the efficiency scores.

**Results:**

The mean bias-corrected technical efficiency score has improved to 0.75 in 2017 from 0.71 in 2016. In both years, more than 50% of thalassaemia care centres showed good efficiency scores (0.8-1.0). Management factors that affect the efficiency scores include separation of patient management (β = 0.0653) and budget (β = 0.0843), where they are found to positively affect the efficiency scores. In contrast, having longer operating hours is found to inversely influence the performance levels (β = − 0.4023).

**Conclusions:**

The study provides a pioneering framework to evaluate the technical efficiency of thalassaemia treatment centres in public healthcare settings and could provide a useful guide for policymaker and thalassaemia care centre managers to improve efficiency in service delivery to thalassaemia patients and their caregivers without compromising quality of care.

## Background

Healthcare is a significant component of country expenditures. Across the Organization for Economic Co-operation Development (OECD) countries, health spending accounts for almost 10% of a country’s gross domestic product (GDP) (1). Optimizing efficiency has become increasingly critical with the growing demand for finite healthcare resources driven by population growth and an ageing society. Hence, policymakers are urgently finding more efficient ways to deliver health services. Efficiency improvements in healthcare delivery, even in small amounts, can yield considerable savings on resources, which then promotes further expansion of services for the community. Efficiency in healthcare is an attribute of performance that is measured by examining the relationship between specific products of the healthcare system and the resources used to create those products (2, 3). A provider is said to be efficient if the input could be minimized to produce a set of given outputs or conversely able to maximize the output for a given set of inputs.

Malaysia has one of the most accessible healthcare facilities in the world. The Malaysian healthcare system consists of private and public healthcare systems that coexist in tandem. Private healthcare is largely profit-driven and primarily financed by patients’ out-of-pocket expenses. A vast network of public hospitals, health centres and health clinics serving both urban and rural populations are heavily subsidized by the government through central taxation (4, 5). In any healthcare organization, policymakers face the arduous task of ensuring equitable public access to optimal-quality healthcare.

Thalassemia is a complex inherited blood disorder with significant prevalence in Asia. It is estimated that more than 300 million people are carriers of this haemoglobin disorder, and the majority of them live in the Southeast Asia region (6). As reported in 2014, there were 6088 cases of thalassemia in Malaysia. The common types found in Malaysia are α or β thalassemia. Phenotypically, thalassaemia varies in its clinical severity and blood transfusion requirement need, and thalassaemia can be further classified into transfusion-dependent thalassemia (TDT) or nontransfusion-dependent thalassemia (NTDT). Haemopoietic stem cell transplantation is the curative treatment for thalassaemia (7).

In Malaysia, however, the choice for transplantation is not widely available; thus, the majority of patients depend on regular blood transfusion (8). Consequently, chronically transfused thalassemia patients are unable to eliminate the excess iron that was released from the breakdown of transfused red blood cells. Hence, excess iron will be deposited in different organs, such as hemosiderin and ferritin, particularly in the heart and liver (9). The accumulation of toxic levels of iron will ultimately lead to complications such as heart failure, diabetes, hypothyroidism and liver disease that increase morbidity and mortality rates in thalassaemia patients. Iron-chelating drugs, therefore, are vital to these regular blood recipients to prevent the effects of iron accumulation.

Studies have shown that Malaysia has one of the most well-established quality medical care programs, including screening programs, counseling, curative care and palliative care for thalassemia patients (10, 11). Thalassaemia patients in Malaysia almost entirely seek treatment in government-funded public hospitals. TDT patients require regular transfusion once every three to four weeks, whereas NTDT patients need transfusion after a longer period depending on their clinical symptoms. This means that a significant number of patients need to visit thalassaemia care centre (TCC) in public hospitals on a monthly basis. TCC is fundamentally a centre that focuses on the management of thalassaemia patients. It operates as part of a hospital department and varies between hospitals and is managed either under paediatric or haematology specialty.

As time goes by, the number of thalassaemics continues to rise despite numerous awareness campaigns and efforts from governmental bodies and advocate groups. The limited availability of prenatal diagnosis and the apprehensive public sentiment on selective abortion for fetuses in Malaysia contribute to an increasing number of thalassaemics (12). This in the end put substantial strain on public healthcare, as thalassemia patients require lifetime healthcare and monitoring (7).

To the best of our knowledge, no study has explored the efficiency of thalassaemia treatment prior to this study. Thalassaemia patients, as we know, are bound lifelong to regular hospital visits and thus bring about high utilization of public healthcare resources. Malaysia, with a high number of thalassaemics as well as its unique healthcare system, will serve as a good foundation for further exploration in thalassaemia care centres.

This study aims to evaluate the technical efficiency of thalassaemia care centres throughout Malaysia and the management factors that affect the efficiency. This paper is organized into several parts. Section 2 discusses related previous efficiency studies in healthcare. The methodology of the study and details of the selection of input/output, model and determinants influencing efficiency scores are addressed in Section 3. The remainder of Sections 4 and 5 discuss the findings and policy implications.

## Insights from the literature on efficiency studies

Numerous studies on healthcare efficiency and hospitals have been conducted over the years using DEA. Some selected studies measured hospital efficiency using DEA in Saudi Arabia (13), South Korea (14), Turkey (15), Greek (16) and Gambia (17). Several other studies adopted DEA to compute efficiency in regard to disease conditions such as stroke (18), diabetes (19), malaria (20), and HIV/AIDS (21). In addition, the application of DEA has also been extended to measure the efficiency of healthcare services, such as oral health services (22), nursing units (23), primary care (24), maternal health (25, 26), pharmacy services (27, 28) and general practitioners (29). Additionally, there are several comprehensive reviews on efficiency studies, such as by Kohl et al. (30), Hollingsworth et al. (31), and O’Neill (32). A similar point of issue raised by all the review papers was aimed at translating the findings to address real practice issues and bridging the gap of theoretical postulation into real value for policymakers and managers.

While all of the stated studies adapt the traditional two-stage DEA, we also found several other studies that utilize double bootstrap DEA to assess efficiency in healthcare, such as by Caavalieri et al. (33), Hamzah and See (34), See and Yen (35), Chowdhury et al. (36) and Staat et al. (37). Simar and Wilson (38) introduced double bootstrap DEA to address the issues of the data generating process during efficiency score calculation as well as second-stage regression to investigate the environmental factors affecting efficiency scores.

Although DEA is considered the preferred method, it is still worth mentioning some hospital-associated efficiency studies in which stochastic frontier analysis (SFA) was applied. Some example of studies include Goudarzi et al. (39) looking at the efficiency of teaching hospitals in Iran, Yildiz et al. (40) assessing the technical efficiency of Turkish hospitals, Canadian hospital efficiency study by Abeney et al. (41) and Rosko et al. (42) a review paper discussing SFA applied efficiency studies on 27 hospitals in the United States.

As discussed, there has not been any study looking at the technical efficiency of thalassaemia care centres. However, if we based the process and functionality of similar practice or service, the most relatable would be of a dialysis centre. The rationale behind this is because the dialysis centre runs as outpatient or ambulatory care where the patient comes to be dialyzed. The cluster patients attending the facilities are mostly registered patients, which are similar in the case of thalassaemia.

On that note, several efficiency evaluation studies have been conducted in dialysis centres. A paper by Gerard and Roderick (43) evaluated the efficiency of 82 haemodialysis satellite units in England and Wales using an output-oriented variable returns to scale (VRS) DEA model. The inputs selected were the number of nurses and dialysis machines, while the selected output was patients treated per week. The results from the study showed that the mean overall efficiency score was 0.94. Twenty-four units were identified as best practicing units in which 10% more output was to be augmented if all units were to be efficient.

Additionally, Kontodimopuolos et al. (44) applied DEA to assess efficiency on 118 haemodialysis units in Greece. Selected inputs were the number of nurses and dialysis machines. Input-oriented VRS DEA model were selected in the study. The study found that units should reduce input utilization by 30% to achieve efficiency. The study also found that being in the private or public sector affects the efficiency scores.

A study by Ozgen and Ozcan (45) applied input-oriented VRS DEA to assess the efficiency of 840 freestanding dialysis facilities in the United States (U.S.). The input variables selected include labor (doctors and nurses), capital input (the dialysis machine) and operating costs (supply, drug, laboratory and maintenance costs). Some of the influencing factors assessed were market competition, reusability of dialyzer, affiliation with the size of the facility chain, ownership nature of the centre and interaction with other forms of facility (nonprofit, for-profit, government-owned). The study showed that almost 22% of facilities are technically efficient, with an average score of 0.79, and inefficient providers could have reduced their input by 21% to achieve full efficiency. Analysis proved that the technical efficiency scores are affected by ownership form, affiliation with dialysis systems of different sizes, and operating in mixed-ownership markets.

Subsequently, following the study by Ozgen and Ozcan (45), Shreay et al. (46) expanded and evaluated 4343 freestanding haemodialysis units. The findings showed that a quarter of units operate at full efficiency with a mean score of 0.78, which is similar to previous studies. Shreay et al. (46) also found that input reduction by 30% would aid inefficiency units in reaching the efficient frontier. Findings from the study showed that region, organization size and urban location influenced the efficiency scores. Consistent with the findings of Ozgen and Ozcan (45), the ownership form was found to affect the score. Apart from that, geographic region and urbanicity also influence the efficiency scores of the study units.

Several other studies compare the efficiency of departments of wards in hospitals. This included a study by Foo et al. (47) comparing the efficiency of ophthalmology services in public hospitals. Labour inputs, such as ophthalmologists, nurses and assistant medical officers, and the output of the number of surgeries conducted and inpatient and outpatient cases were selected. It was found that 33% of ophthalmology departments scored good efficiency scores. In addition, a study in Turkey compared the different clinical departments in a university hospital. Inputs analyzed include the number of beds, drug expenditure, and number of faculty members whilst the output variables are the number of outpatients and inpatients. The findings showed that the orthopaedic department was the most efficient (48).

Mark et al. (23) studied the technical efficiency of 118 acute care nursing units. The inputs selected were the number of registered nurses, operating expenses and number of beds, and outputs were the number of discharges, patient satisfaction as a quality measure and rates of medication error as a measure of patient safety. Sixty percent of units evaluated were found to operate at less than full efficiency. Improvement suggestions include reducing labour hours and reducing medication errors (23). Two other studies assessed the efficiency of pharmacy services in hospitals. Nineteen percent of hospital pharmacies operate at full efficiencies. The input selected was the number of pharmacists and support personnel, while outputs were drug dispensed, drug purchase and patient-oriented services (28). Another study focusing on pharmacy services is Hamzah and See (34), in which 124 hospital pharmacies were evaluated. This study investigates the effect of hospital size on technical efficiency scores and found that small hospitals scored better in technical efficiency scores than larger and medium hospitals.

Findings from these studies demonstrated the usefulness of DEA in addressing issues of resource allocation and output optimization by assessing efficiency levels while capturing the complexity and managerial trade-offs that characterize healthcare services.

## Methods and data

This is a multicentre study on the technical efficiency of thalassaemia treatment centres in public hospitals in Malaysia.

### Efficiency measurements

DEA is a nonparametric technique where efficiency is expressed as the ratio of sums of the weighted outputs to the sums of the weighted inputs. DEA measures technical efficiency by first identifying a ‘best practice’ production possibility frontier based on decision-making units (DMUs) achieving the highest output mix for their input levels. These DMUs lying on the frontier would be considered efficient compared to others in the sample and would be assigned a score of one. Other DMUs would then be benchmarked against these frontier centres most similar to themselves (the ‘peers’) and then be given scores of less than one by comparing their output/input ratio (49).

Some of the main approaches to evaluate efficiencies are SFA and DEA. While both approaches provide comprehensive quantification of performance, there are still some limitations. One of the main disadvantages of SFA is that they require predetermination of the functional form of the production frontier as well as the need for a large sample size. On the other hand, DEA, with its deterministic property, does not account for the measurement of error in computing the efficiency measures.

DEA is selected for this study because it allows for multiple inputs and outputs to be used to compute a single efficiency score for each DMU. DEA also identifies the best performance by DMU rather than the averages. This allows derivation of various performance indicators and identification of peers most relevant to each DMU for mutual learning. DEA is able to handle noncommensurate input and outputs without having to put unit prices for each. Apart from exploiting all the advantages of DEA, this study also adopts DEA due to the relatively small number of samples.

#### Standard DEA model

DEA model analysis can use either input- or output-oriented approach. In the input-oriented model, the calculation of technical efficiency is computed with emphasis on the proportional reduction of inputs while maintaining the same level of outputs. On the other hand, output-oriented models tend to proportionally augment outputs. In this study, an input-oriented model is chosen because the output measures (i.e., blood transfusion) are inflexible and highly dependent on patients’ clinical condition. Apart from that, most of the input variables are under the oversight of each DMU.

This study also assumes VRS DEA to measure technical efficiency (TE) scores. VRS is one type of various models to be used based on the scale of operation of the DMU. This model assumes varying returns to scale, which allows the best practice level of outputs to inputs to vary according to the size of the DMU operating. VRS is less restrictive than the constant returns to scale (CRS) model, where the assumption is that the returns of outputs to inputs are proportionately constant or linear.

To describe the standard DEA model, the notation X represents the K × N matrix of inputs, consisting of K inputs from N hospital TCCs, while Y represents the M × N matrix of outputs, consisting of M outputs from N hospital TCCs. The input-oriented CRS DEA model is summarized as follows:

1 $$ {\mathit{\operatorname{Min}}}_{\varnothing, \uplambda}\varnothing $$

Subject to:

$$ -{y}_i+Y\uplambda \ge 0, $$

$$ \varnothing {x}_i-X\uplambda \ge 0 $$

$$ \uplambda \ge 0 $$

∅ signifies a scalar, while λ denotes an N × 1 vector of constants. The resulting value of ∅ is the efficiency score for the i^th^ Thalassaemia treatment centre, where 1 − ∅ is the proportional input reduction that is attained by i^th^ TCC given the output level. The CRS output model can be expanded to VRS with the assumption that each DMU has variable economies of scale. In VRS, convexity constraint *N*1^′^λ = 1 is added in the equation. Input-oriented VRS model is expressed as follows:

2 $$ {\mathit{\operatorname{Min}}}_{\varnothing, \uplambda}\varnothing $$

Subject to:

$$ -{y}_i+Y\uplambda \ge 0, $$

$$ \varnothing {x}_i-X\uplambda \ge 0 $$

$$ N{1}^{\prime}\uplambda =1 $$

$$ \uplambda \ge 0 $$

where *N*1 is an *N*1 × 1 vector of ones while the other parameters are as defined above. The value of scale efficiency (SE) can then be derived from either CRS TE or VRS TE (50). Therefore, the SE score can be calculated by the following equation:

3 $$ {SE}_I={TE}_{I, CRS}/{TE}_{I, VRS} $$

#### Double bootstrap DEA approach

As mentioned above, one of the weaknesses of DEA is that it lacks statistical properties. The introduction of a bootstrap approach to calculate efficiency scores would allow DEA to compensate for the lack of statistical properties. Simar and Wilson (38) showed that there is a serial correlation between the efficiency scores. Thus, the traditional utilization of second-stage regression will violate the basic assumptions of the regression model. This issue is primarily because the generated efficiency scores are an index of relative efficiency instead of an absolute efficiency index.

Simar and Wilson (2007) criticized the Tobit regression used in the second stage and instead proposed double bootstrapping truncated regressions. A double bootstrap DEA approach employed a ‘sensible’ data generating process and parametric bootstrap procedure which is a better option as compared to classical DEA approach. This is because there is a lack of a well-defined data generating process and misleading inference in a conventional approach. The bootstrap approach could also be extended to measure the bearing of environmental factors influencing the efficiency scores, as proposed by Simar and Wilson (38). This enables a more consistent inference during the second-stage regression while simultaneously constructing confidence intervals and generating standard errors for the DEA-efficiency scores. This approach is detailed in following paragraphs.

In the first stage, TE scores are calculated through the DEA model, which will then be regressed against a set of explanatory variables as summarized below:

4 $$ {\varnothing}_m=a+\beta {z}_m+{\varepsilon}_m $$

where ∅_*m*_ is the TE score and *z*_*m*_ is the vector of variables that influences the technical efficiency scores of thalassaemia treatment centres. Β is the vector of the parameters to be estimated, and *ε*_*m*_ is the error term under the assumption of being conditional on *z*_*m*_ and independently distributed as a truncated normal distribution. This second-stage analysis is instrumental to provide insight into the influence of explanatory variables on the TE scores of each DMU. This in turn helps policymakers initiate changes to improve efficiency.

**Fig. 1:**
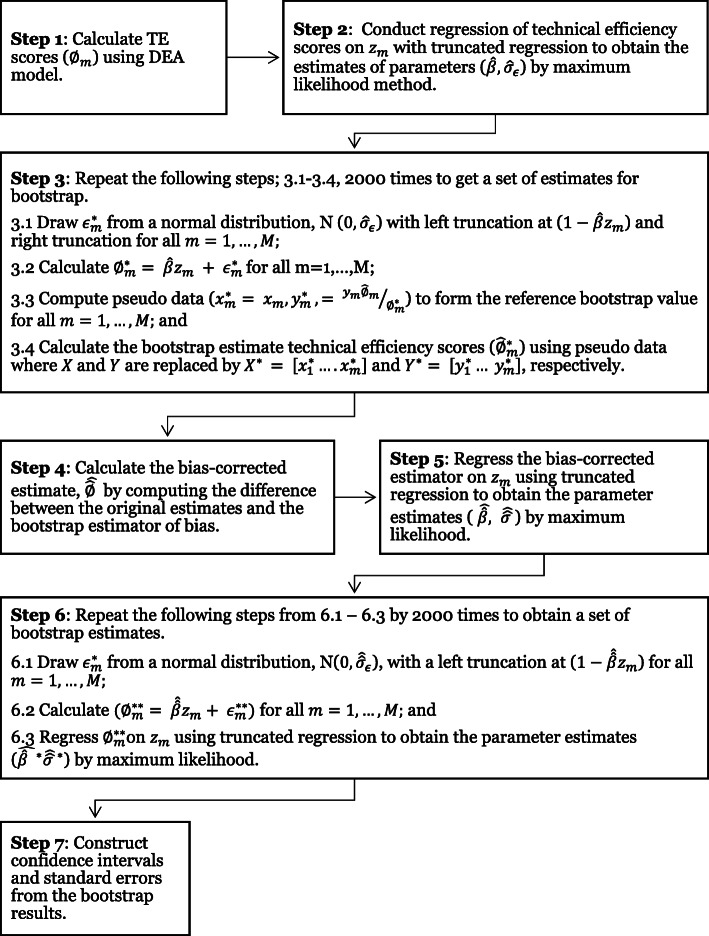
Step by step double bootstrap DEA approach

Detailed algorithm #2, as proposed by Simar and Wilson (38), for this approach is as illustrated in Fig. 1.

### Sample and data collection

Secondary data from 30 public hospitals over the period of two years (2016–2017) were collected from hospital records and the Malaysia Thalassaemia Registry. This study was approved by the Malaysia Medical Research and Ethics Review Committee (NMRR-17-2614-38,966 (ISR)).

#### Selection of input and output measures

**Fig. 2:**
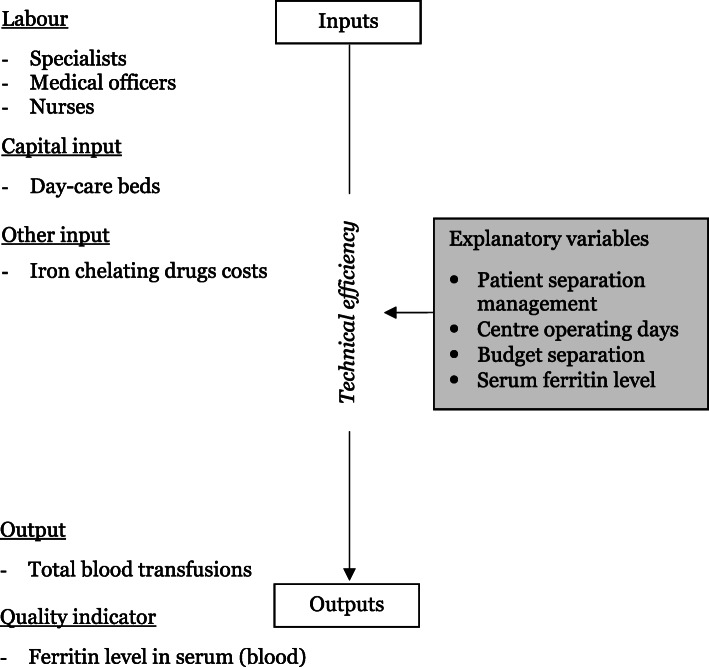
Relationship of input to output and explanatory variables affecting technical efficiency scores

In this study, the workflow description of TCCs providing regular blood transfusions to patients was observed and identified. This is done to select the appropriate input and output measures and to balance the clinical outcome and availability of data. The selected input and output measures must capture the treatment intent to maintain the well-being of the patient and prevent complications from occurring. Other than surviving into adulthood, patients should be able to carry out usual daily activities with an acceptable quality of life during their period of life. Selection of both input and output variables is essential to be reflective of the entire process of care delivery to thalassaemia patients (Table 1). An illustration of the relationship of selected inputs to outputs and the explanatory variables is presented in Fig. 2.

**Table 1:** Input and output variables used in the analysis

Variables	Unit	Data source
Inputs		
Number of FTE doctors (specialists and medical officers)	Headcount	Survey
Number of FTE nurses	Headcount	Survey
Day-care beds	Number	Survey
Drug costs	MYR	Survey
Outputs		
Total blood transfusions	Frequency	MTR
Number of patients achieving blood target level of ferritin	Percentage	MTR

Notation: FTE, denotes full-time equivalent; MYR, denotes Malaysian Ringgit; MTR, denotes Malaysia Thalassaemia Registry.

Generally, blood transfusions required by thalassaemia patients are conducted in day-care units in hospitals. Adult and paediatric patients are usually managed by different department – haematology or paediatric – in a separate area. However, in some TCCs, both adult and paediatric patients are managed and treated together in a common place (combined day care). Transfusing patients are attended by nurses while being monitored and clinically assessed by medical doctors. During this episode of care, the patient will be assigned a day-care bed to rest during the transfusion process. The serum ferritin level in patients is one of the outcome measures to determine the risk of iron overload complications. The patient will be prescribed iron chelating drugs based on clinical assessment by doctors.

##### Input measures

Input measures are chosen based on previous literature on hospital efficiencies (37, 51–53). Common input measures used are labour; number of staff, capital; hospital beds, and drug costs (54). Apart from previous literature as a guide in input selection, the final list of inputs was also discussed and agreed upon by organization managers to ensure that all selection would be able to represent actual resource consumption during the process of flow-in-care delivery to thalassaemia patients.

##### Labour

The most essential resources in every TCC to provide treatment to thalassaemia patients are the staff – doctors and nurses. This is supported by various studies on hospital efficiency where doctors and nurses were included as labour input (53). The most accurate measurement of labour input would be total hours worked grouped into level of skills and wages (55). Unfortunately, in this study, information on total work hours is unavailable. Hence, data on the number of full-time-equivalent staff members were selected as a proxy. In each TCC nationwide, there are two categories of doctors, namely, specialists and medical officers. The wage of doctors varies according to qualification. The wages in each category, nonetheless, are similar across the country, as employment authorities are under the purview of the Ministry of Health (MOH) central administration. For the purpose of analysis, the wage ratio was used to adjust all doctors into one group. For nurses, the number of nurses working in TCCs was selected.

##### Day-care beds

Most healthcare efficiency studies, such as those by Staat et al. (37) and Chaabouni et al. (52) utilize simple measures of overall physical measures of capital used by each healthcare facility. As shown in numerous studies, tangible assets such as the number of beds are used as proxies to assess capital input. In most hospitals, patients receive blood transfusion in TCCs that are integrated with day-care units. As thalassaemia is a lifelong medical condition, some patients require periodical but continuous blood transfusion over their lifetime.

##### Drug costs

Drug expenditure must be selected as production input, as thalassaemia patients receiving chronic transfusion will require iron-chelating drugs to prevent iron overload-related complications. In Malaysian public hospitals, all medications for thalassaemia patients are supplied gratuitously to patients, as the cost is entirely borne by the public hospital (4). Therefore, the total drug cost of iron chelating agents is a good selection as an input variable to demonstrate the cost burden.

#### Output measures

As mentioned before, there has not been any efficiency study in regard to thalassaemia before; thus, due to the lack of literature references, two selected output measures are chosen to represent the primary activity in all TCCs and to best reflect the treatment intent of the entire process of care.

##### Total blood transfusions

In all hospitals, the primary operational function of TCCs is to provide blood transfusion to thalassaemia patients, both TDT and NTDT. Total blood transfusions were selected as output, as this would be the reflection of the amount of service delivered to attending patients in each DMU and serve as a proxy to represent the number of patients in each TCC.

##### Total patient achieving the target serum ferritin level

Thalassaemia patients on regular transfusion are monitored for iron overload, which may cause toxicity and ultimately lead to complications. As described in clinical practice guidelines, ensuring the serum ferritin level over a period of time to be below 2500 μL is a good estimate of the risk of iron overload complications and subsequently improves survival (56). In this study, the total number of patients achieving the target serum ferritin was used as a desirable outcome of thalassaemia treatment.

#### Explanatory variables

To further explore the determinants that affect TCC efficiency scores, four explanatory variables that were related to management factors and specific characteristics are selected primarily following discussion with subject matter experts and policymakers. As there has not been previous similar literature focusing on the operation of thalassaemia care centres, literature searches were only conducted to ensure relevance to the selected determinants, as detailed in the following discussion.

### Patient separation management (MANAGEMENT)

As mentioned above, the management of blood transfusion for thalassaemia patients in certain TCCs is separated into adult or paediatric patients, whereas some are combined and managed together in the same day-care unit and same healthcare team. Cappellini et al. (7), a thalassaemia care centre, ideally should separate care for adults and paediatric patients. It is expected that separated management would increase the efficiency of the TCC and thus give a positive coefficient.

### Centre operating days (OPERATING)

The decentralization of management to hospital administrators also creates variation in the number of operating days in each TCC. The number of operating days is generally believed to have an implication on the technical efficiency of each DMU. TCCs that operate longer will give convenience to patients to attend TCCs and will be more effective in achieving the desired treatment output. Therefore, the longer the centre operates, the higher the efficiency of the centre. In this study, the number of days a TCC operates is weighted relative to the maximum operating days for DMUs.

### Budget separation (BUDGET)

Funding of all public hospitals is sourced from the central MOH. The detailed budget for each specialty is then determined by the state health governing body. In some hospitals, the thalassaemia-specific budget is determined and centralized at the state level, while in some other hospitals, the authority is decentralized to hospital administrators to determine the necessary budget for thalassaemia care in that hospital. Once decentralized, the budget will be aggregated and given as lump sum to the related departments via hospital administration. Having centralized budgeting helps TCCs, despite their size and geographical location, better conduct procurement on thalassaemia care-related expenditures. Budget centralization application is conceivable in thalassaemia management, as the patients involved are of a known cluster. The disadvantage, however, will be less flexibility as to how a department does their spending. Consequently, a centralized where budget for thalassaemia is separated distinctively would positively influence the efficiency of the centre.

### Serum ferritin level (COMPLICATION)

The treatment goal of thalassaemia treatment as described by clinical guidelines is principally to prolong survival and reduce the risk of complications while simultaneously ensuring optimal quality of life for thalassemia patients. As mentioned above, patients who receive regular blood transfusion are highly susceptible to iron overload complications. Serum ferritin level monitoring is a valuable tool for the risk of complications from blood transfusions, as the association of prognosis and control of serum ferritin has been established (7). The proportion of patients monitored for serum ferritin level who achieved optimal target level for each DMU was used to regress against the efficiency scores. It is widely accepted that urban-located and larger hospitals have more complicated cases due to facilities and expertise available in the facilities. A higher number of cases with complications is believed to have a negative impact on the efficiency of TCCs.

### Technical efficiency scores over a two-year period (YEAR)

This study collected two-year data from all TCCs. The difference in the technical efficiency scores over the two-year period was regressed to establish if there was any effect on the TE scores.

## Results and discussion

Data from 30 TCCs in two consecutive years from 2016 to 2017 were analyzed. The descriptive statistics and efficiency scores are shown in Table 2 and Table 3, respectively. Subsequently, the results of second-stage bootstrap truncated regressions are presented and discussed.

**Table 2:** *Descriptive statistics for the selected input and output variables for 2016 and 2017*

Measures	2016				2017			
	Mean	SD	Minimum	Maximum	Mean	SD	Minimum	Maximum
Inputs								
Number of doctors	5.87	3.27	1.00	13.54	6.68	4.24	1.00	17.13
Number of nurses	5.30	3.84	1.00	14.00	5.47	4.10	1.00	16.00
Number of day-care beds	13.93	6.60	3.00	27.00	14.43	7.48	3.00	36.00
Drug costs (MYR)	802,797.70	880,995.60	17,783.69	4,279,149.00	1,185,664.00	1,137,297.00	5195.12	4,741,593.00
Outputs								
Total blood transfusions	462.27	462.71	21.00	1968.00	608.40	581.49	11.00	2507.00
Total patients achieving blood target level of ferritin	30.77	29.95	2.00	116.00	35.97	31.83	2.00	111.00

**Table 3:** *Original DEA efficiency scores, bias and bias-corrected efficiency scores*

Score	2016				2017			
	Mean	SD	N	%	Mean	SD	N	%
Original TE score, Mean (SD)	0.79	0.20			0.83	0.19		
Original TE score (Full efficiency 1.00), N (%)			9	30.0			10	33.3
Original TE Score 0.80–1.00, N (%)			9	30.0			10	33.3
Original TE Score 0.60–0.79, N (%)			5	16.7			5	16.7
Original TE Score < 0.60, N (%)			7	23.3			5	16.7
Bias-corrected TE score, Mean (SD)	0.71	0.16			0.75	0.16		
Bias-corrected TE score 0.80–1.00, N (%)			15	50.0			16	53.3
Bias-corrected TE score 0.60–0.79, N (%)			7	23.3			9	30.0
Bias-corrected TE score < 0.60, N (%)			8	26.7			5	16.7

### Descriptive statistics

Descriptive statistics for selected inputs and outputs are presented in Table 2. The efficiency score is computed based on a four-input and two-output model. The mean for each input and output variable included in this study is presented for both years. Referring to Table 2, thalassaemia care centres on average spent almost 1.2 million Malaysian Ringgit on iron-chelating drugs, and more than 600 blood transfusions to thalassaemia patients took place in 2017.

### Original DEA efficiency scores and bias-corrected efficiency scores

Mean original TE score and mean bias-corrected efficiency scores and distribution are tabulated as in Table 3. The efficiency scores generated are then categorized into four levels: full efficiency (TE score of 1.00) followed by good (TE score of 0.80–1.00), average (TE score of 0.60–0.79) and poor (TE score < 0.60). The categorization of the scoring was decided by focus group discussion involving subject matter experts. This was further strengthened by two studies by Hamzah and See (34) and Rattanachotphanit et al. (28) applying similar scoring classifications. Of all the DMUs in 2016, the mean original score was 0.79, with 30.0% achieving full efficiency and 23.3% of DMUs scoring poorly. In 2017, the mean original score slightly improved to 0.83, with 33.3% performed at full efficiency. After applying bootstrap methodology, the mean TE score (bias-corrected TE score) in 2016 exhibited a lower value and smaller slightly smaller distribution at 0.71 and 0.16, respectively. The distribution of DMU efficiency levels is also altered as more DMUs fall into a good efficiency level. The mean bias-corrected score showed slight improvement, with 0.75 and a distribution of 0.16. In this study, bias-corrected TE scores were more accurate, as they fell within the estimated 95% confidence interval and showed more robustness. From the calculated TE scores, it can generally be implied that DMUs can achieve full efficiency by reducing their input by 29% in 2016 and 25% in 2017.

### Bootstrap truncated regression

Second-stage bootstrap truncated regression was used to further explore the management determinants affecting the technical efficiency of the DMUs. Referring to Table 4, there is an association of a separate management team (MANAGEMENT) in DMUs with the level of efficiency (p value ≤.1). This variable is included to look at the effect of different setting practices of TCCs included as study sites. Several sites combine TCC with adult and paediatric patients, while the remaining hospital has separate TCC for adult and paediatric thalassaemia patients. As the result showed a positive coefficient (β = 0.065), this suggests that management separation of thalassaemia patients would positively impact the technical efficiency level of a DMU. This practice of separation is in parallel with what is recommended, as it will promote multidisciplinary thalassaemia care apart from providing safety and privacy to patients (7, 57).

**Table 4:** *Result of bootstrap truncated regression*

Variable ^a^	Bootstrap coefficient	Bootstrap Std. Err.	95% Bootstrap CI^b^	
			Lower	Upper
MANAGEMENT	0.0653*	0.0394	−0.0086	0.1417
OPERATING	−0.4023***	0.0862	−0.5799	−0.2351
BUDGET	0.0843***	0.0357	0.0152	0.1548
COMPLICATION	−0.0169	0.1223	−0.2564	0.2228
YEAR	0.0593	0.0347	−0.0084	0.1272

Intercept is included in the bootstrap truncated regression model; CI: Confidence intervals

^a^ Dependent variable: Bias-corrected efficiency scores

^b^ Figures are computed using 2000 bootstrap interactions

*** and * represent statistical significance at 1 and 10%, respectively

Another determinant that showed a strong relationship was the centre operating days (OPERATING). Interestingly, the coefficient (β = − 0.4023, *p* value <.001) of the operating days is computed to be negative, which implies that the efficiency level decreases as the number of days a TCC operates increases. In short, operating for longer hours may not improve the efficiency of the facility. The possibility is that there is an inefficient allocation of resources such as labour if TCCs operate for longer days. Unlike any other departments or services in hospitals, TCCs are unique in that all patients are already registered and known. This relates to the fact that thalassaemia patients are regular and long-term patients frequenting each TCC. Therefore, as the solution, a centre could adjust and tailor-make the operating day and time accordingly.

Apart from that, special allocation of budget for thalassaemia (BUDGET) at the hospital level was found to have a positive relationship with the technical efficiency score (β = 0.084, *p* value <.05). This means that separate allocation for thalassaemia will help a TCC operate more efficiently. There are various practices in budgeting for thalassaemia programmes at each state in Malaysia. This finding showed that it is best to separate a specific budget for thalassaemia higher than the state level.

The variable COMPLICATION represents one perspective of treatment effectiveness for each TCC by which the patient’s serum ferritin is monitored and maintained at the target level. Serum ferritin levels serve as a good indicator for the prognosis and risk of complications associated with thalassaemia. The result from bootstrap truncated regression showed that the variable COMPLICATION was not statistically significant (*p* = 0.89) in affecting TE scores. Hence, from this result, it can be implied that although certain TCCs treat more complicated cases than the rest, the TE score is independent of this factor. Finally, as this study takes into account data from a period of two years from all the centres, the data collected for the two-year period were regressed (YEAR). However, the results showed no significant difference (*p* = 0.09) in technical efficiency scores over the two-year period.

## Policy implication and conclusion

The translational value of each efficiency study conducted has always been of interesting discussion among policymakers. In this study, we envision initiating changes to improve policy that consequently enhance healthcare service delivery to patients as the focal point in any healthcare system.

As discussed previously, thalassaemia has increasingly become a pressing public health issue in Malaysia that necessitates swift action to improve the efficiency and resource allocation of thalassaemia care centres in Malaysia. As mentioned above, to the best of our knowledge, no other study has explored the efficiency of thalassaemia centres. This study hopes to set an antecedent on the framework for efficiency analysis of thalassaemia treatment.

The study results demonstrated that more than 50% of DMUs were performing at a good technical efficiency level in the two years of data collection. Consequently, for the remaining DMUs, efficiency can be achieved if they were to reduce their input resources by between 25 and 29%. This could be attained either by reducing their scale of operation by means of reallocating staff involved or by reassessing the expenditure on the purported iron chelating medication. As the selection of medication is dependent on patients’ clinical assessment, other measures, such as diversifying the procurement of iron chelators for alternative brands with lower price, should be taken.

One of the investigated determinants that affects the efficiency of a DMU is the separation management of adult and paediatric thalassaemia patients. The practice of having adult patients transfused alongside pediatrics, although not uncommon, as evident by several TCCs studied, poses some disadvantages. This practice may be justified if the patient pool is small for the centre, but with a higher number of patients, a TCC manager should work towards separate and dedicated yet not isolated services and space for adults and paediatric patients. This separation was advocated by clinical practice guidelines for transfusion-dependent thalassaemia (7). The separation would encourage better patient management, as both adults and pediatric patients require different medical needs apart from ensuring safety and privacy to patients (57).

Another interesting finding for a TCC to improve efficiency is re-examining their operating days. TDT thalassaemia patients require frequent TCC visits due to the need for preparation for transfusion and actual blood transfusion until review by various medical specializations. As reported in studies, patients and caregivers complain of high productivity loss due to absence from work to attend treatments and visits (58, 59). Adhering to follow-ups and visits is highly time-consuming itself, which is further worsened as it is impeded by the operating hours of a TCC. Some centres operate at longer days to give flexibility to the patients and guardians to come for blood transfusion on days or hours that suits their schooling or working hours. This flexibility, although simple, affects the time gained for them to be in school and employability for adult thalassaemia patients.

As the results showed, the efficiency level of TCCs is inversely related to the operating days, which means that TCCs would be less efficient with a higher number of days they operate. However, the efficient use of TCCs must be maximized without compromising patient care and needs. As discussed, reducing the operating days could cause inconvenience to patients having to abide to restrict the operating day. As a reconciliation, TCC managers should consider having discussion for shared decision making with patients to determine the most suitable operating days and hours without also conceding on the wellbeing of staff of the centre (7). This solution is in line with the WHO’s Innovative Care for Chronic Conditions (ICCC) framework recommending self-management and changes to the organization of care delivery with active participation from both patients and healthcare teams (60).

Malaysia, a heavily subsidized healthcare system from the public coffer and with ever-finite resources, makes it imperative to have a precise and meticulously curated budget mechanism. Budget separation of having to allocate a specific budget for thalassaemia management is another possibility to improve efficiency. The common current practice is that the budget for thalassaemia is decentralized to departments at the hospital level. It may be more favourable in terms of efficiency if the budgeting is centralized to a higher level, such as the state or federal level. Having separate funding for a disease-specific or issue-specific budget is favourable to achieve targeted treatment targets (61).

Nonetheless, DEA is a data-driven deterministic tool to measure efficiency and is not invincible of limitations. The output measures selected cannot be considered ideal for thalassaemia. Variables that could reflect complications from thalassemia would be an excellent indicator of treatment quality for thalassaemia care in Malaysia. As this study is sourced by a database, it is limited by the quality of data collected and recorded in the databases. To account for these issues, future studies should include more comprehensive output measures that reflect the complexity of thalassaemia care. Despite the limitations, this study provides a pioneering framework to evaluate the TE of thalassaemia treatment centres in public healthcare settings and could provide a useful guide for policymakers and TCC managers to improve efficiency in service delivery to thalassaemia patients and their caregivers without compromising quality of care.

## Data Availability

The datasets generated and analyzed during this study are not publicly available due to confidentiality nature of the data.

## Abbreviations

DEA: data envelopment analysis
OECD: Organization for Economic Co-operation Development
GDP: gross domestic product
TDT: transfusion dependent thalassaemia
NTDT: nontransfusion dependent thalassaemia
TCC: thalassaemia care centre
SFA: stochastic frontier analysis
VRS: variable returns to scale
DMU: decision making unit
TE: technical efficiency
CRS: constant returns to scale
SE: scale efficiency
